# Levels of inflammatory cytokines MCP-1, CCL4, and PD-L1 in CSF differentiate idiopathic normal pressure hydrocephalus from neurodegenerative diseases

**DOI:** 10.1186/s12987-023-00472-x

**Published:** 2023-10-13

**Authors:** Madelene Braun, Gustaf Boström, Martin Ingelsson, Lena Kilander, Malin Löwenmark, Dag Nyholm, Joachim Burman, Valter Niemelä, Eva Freyhult, Kim Kultima, Johan Virhammar

**Affiliations:** 1 Department of Medical Sciences, Neurology, Uppsala University, Uppsala, Sweden; 2 Department of Public Health and Caring Sciences, Molecular Geriatrics, Rudbeck Laboratory, Uppsala University, Uppsala, Sweden; 3 Centre for Clinical Research, Uppsala University, Västmanland County Hospital, Västerås, Sweden; 4 Krembil Brain Institute, University Health Network, Toronto, Ontario Canada; 5 Tanz Centre for Research in Neurodegenerative Diseases, Departments of Medicine and Laboratory Medicine & Pathobiology, University of Toronto, Toronto, Ontario Canada; 6 Department of Cell and Molecular Biology, Uppsala University, Uppsala, Sweden; 7 Department of Medical Sciences, Clinical Chemistry, Uppsala University, Uppsala, Sweden

**Keywords:** Normal pressure hydrocephalus, MCP-1, CCL4, PD-L1, Biomarkers, Cerebrospinal fluid, Neuroinflammation, Proteomics

## Abstract

**Background:**

Neuroinflammatory processes have been suggested to play a role in the pathophysiology of neurodegenerative diseases and post-hemorrhagic hydrocephalus, but have rarely been investigated in patients with idiopathic normal pressure hydrocephalus (iNPH). The aim of this study was to investigate whether levels of inflammatory proteins in CSF are different in iNPH compared to healthy controls and patients with selected neurodegenerative disorders, and whether any of these markers can aid in the differential diagnosis of iNPH.

**Methods:**

Lumbar CSF was collected from 172 patients from a single center and represented iNPH (n = 74), Alzheimer’s disease (AD) (n = 21), mild cognitive impairment (MCI) due to AD (n = 21), stable MCI (n = 22), frontotemporal dementia (n = 13), and healthy controls (HC) (n = 21). Levels of 92 inflammatory proteins were analyzed using a proximity extension assay. As a first step, differences between iNPH and HC were investigated, and proteins that differed between iNPH and HC were then compared with those from the other groups. The linear regressions were adjusted for age, sex, and plate number.

**Results:**

Three proteins showed higher (MCP-1, p = 0.0013; CCL4, p = 0.0008; CCL11, p = 0.0022) and one lower (PD-L1, p = 0.0051) levels in patients with iNPH compared to HC. MCP-1 was then found to be higher in iNPH than in all other groups. CCL4 was higher in iNPH than in all other groups, except in MCI due to AD. PD-L1 was lower in iNPH compared to all other groups, except in stable MCI. Levels of CCL11 did not differ between iNPH and the differential diagnoses. In a model based on the four proteins mentioned above, the mean area under the receiver operating characteristic curve used to discriminate between iNPH and the other disorders was 0.91.

**Conclusions:**

The inflammatory cytokines MCP-1 and CCL4 are present at higher—and PD-L1 at lower—levels in iNPH than in the other investigated diagnoses. These three selected cytokines may have diagnostic potential in the work-up of patients with iNPH.

**Supplementary Information:**

The online version contains supplementary material available at 10.1186/s12987-023-00472-x.

## Introduction

Idiopathic normal pressure hydrocephalus (iNPH) is characterized by gait disturbance, impaired cognition, and urinary incontinence. Treatment consists of shunt surgery, reducing symptoms in 60–80% of patients, while delayed surgery worsens the prognosis [1]. Diagnosis of iNPH can be challenging, as its clinical presentation can mimic various neurodegenerative diseases, and Alzheimer’s disease (AD) comorbidity is common [2]. Diagnostic biomarkers in cerebrospinal fluid (CSF), such as tau and amyloid beta subtypes, are used in the differential diagnostics between iNPH and neurodegenerative diseases at some centers [3–5]. Nevertheless, these markers are insufficient and cannot accurately distinguish iNPH from neurodegenerative diseases or from neurodegenerative comorbidities.

It has been suggested that neuroinflammation plays a role in the pathogenesis of multiple neurodegenerative diseases and hydrocephalus [6, 7]. In posthemorrhagic and postinfectious hydrocephalus, channel hyperactivity is reported in the choroid plexus, where specific receptors and signaling systems initiate an inflammatory response that leads to infiltration of activated inflammatory cells [8]. The choroid plexus functions as the blood–CSF barrier, acting as a gate to immune cell entry into the CNS, and the development of hydrocephalic conditions could be linked to the function of the choroid plexus.

There are many hypotheses surrounding iNPH pathogenesis, including abnormal CSF drainage, cerebral hypoperfusion with secondary hypoxia, and disturbances in the glia-lymphatic (glymphatic) system [8]. Since neuroinflammation has been proposed to be connected to the onset of communicating hydrocephalus of hemorrhagic or other secondary causes and progression of neurodegenerative diseases, we aimed to investigate whether proteins involved in inflammatory processes are altered in CSF in patients with iNPH and whether these proteins can differentiate iNPH from selected neurodegenerative diseases.

## Material and methods

### Patients

This study included a total of 172 patients, distributed as follows: iNPH (n = 74), AD (n = 21), mild cognitive impairment due to AD (MCI/AD) (n = 21), MCI (that did not progress to AD) (n = 22), frontotemporal dementia, FTD (n = 13) and healthy controls (n = 21) (Table 1). Two iNPH patients were excluded due to technical problems with the protein analysis. Sex and age distribution in the groups is demonstrated in Table 1.

**Table 1:** Characteristics of all study participants

	AD	MCI/AD	MCI	FTD	iNPH	C
n	21	21	22	13	72	21
Age, mean (SD)	71 (± 6)	71 (± 5)^†^	72 (± 5)	68 (± 9)^††^	74 (± 6)	82 (± 4)^***^
Sex, Male (%)	11 (52)	10 (48)	11 (50)	9 (69)	48 (67)	12 (57)
T-tau (ng/L), mean (SD)	692 (± 302)^***^ n = 20	620 (± 258)^***^	265 (± 88)^***^	379 (± 163)^***^ n = 4	208 (± 152) n = 71	504 (± 262)^***^ n = 20
Aß-42 (ng/L), mean (SD)	398 (± 90)^†††^ n = 20	428 (± 123)^†††^	826 (± 208)^***^	786 (± 217)^***^ n = 4	593 (± 153) n = 72	787 (± 287)^**^ n = 20

*AD* Alzheimer’s disease, *MCI/AD* mild cognitive impairment due to Alzheimer’s disease, *MCI* mild cognitive impairment, *FTD* frontotemporal dementia, *T-tau* total tau, *Aß-42* Amyloid beta 42. Normal reference ranges: Aß-42: > 530 ng/L; T-tau: < 350 ng/L

Higher than iNPH: * < 0.05; ** < 0.01; *** < 0.001

Lower than iNPH: † < 0.05; †† < 0.01; ††† < 0.001

Patients were diagnosed with iNPH according to international guidelines [9]. They were prospectively and consecutively included in a biobank study (Dnr 2013/278), of which all selected iNPH patients from this study were obtained and included during the period of 2014–2018. Diagnoses of AD, MCI/AD, and MCI were given according to the National Institute on Aging and Alzheimer’s Association criteria (NIA-AA), and an FTD diagnosis was determined according to clinical criteria in combination with neuroimaging [10–12]. Patients in the MCI group were followed for 4–9 years after CSF sampling, and did not convert to AD dementia during that period. Diagnoses and lumbar punctures in the groups with MCI and neurodegenerative disorders were made between 2005 and 2018. The healthy controls were neurologically healthy individuals, free from neurocognitive disorders, and recruited at Uppsala University Hospital through advertisements in a local newspaper. Some of the controls had participated in a previous study [13].

The Swedish Ethical Review Authority approved the study 2018/168, 2021-05439-02, 2013/278, 2005-244, Ö 48-2005 and 2013/187, and informed consent was obtained from all included patients and controls [13].

### Sampling

The CSF samples from all diagnostic groups and controls were collected via lumbar puncture performed at Uppsala University Hospital, Sweden. In the iNPH group, cerebrospinal fluid was collected during a tap test procedure. Samples were stored in polypropylene tubes that were frozen at − 70 °C. For the current analyses, the samples were thawed, aliquoted into microtubes, and refrozen at − 70 °C.

### Proximity extension assay (PEA)

Protein levels were measured with PEA at Olink Proteomics’ laboratory in Uppsala, using the Olink^®^ Inflammation panel (Olink Proteomics AB, Uppsala, Sweden; https://www.olink.com/products/inflammation/, accessed 5 June 2023) according to the manufacturer’s instructions. PEA is an immunoassay with a high sensitivity and specificity, where 1 μl of each sample is analyzed in parallel for 92 protein analytes in a 96-well panel. The method is based on a pair of oligonucleotide-conjugated antibodies that are matched and will attach to each protein. When matching pairs bind to a target protein, hybridization takes place, and a unique model for DNA polymerase dependent extension is created. This produces a PCR sequence that is then amplified. Each protein value in PEA is then measured in a Normalized Protein eXpression (NPX) value. The NPX value is an arbitrary unit on log_2_-scale where a high value corresponds to a higher protein expression (an increase with 1 NPX is a doubled protein concentration). All assay validation data (detection limits, intra- and inter-assay precision data, etc.) are available on the manufacturer’s website (http://www.olink.com).

### Statistics

The selected assay analyzed 92 proteins. Of these, 45 (49%) had detectable values (above the limit of detection, LOD) in all 170 patient samples. A total of 56 proteins (61%) had detectable values for at least 75% of participant samples. Only the proteins with detectable values for at least 75% or more of the patients’ samples were included in further analyses. A principal component analysis (PCA) was used to evaluate patterns of clusters related to the different patient groups and plates.

To limit the number of analyses, iNPH samples were first compared to healthy controls. Only proteins that differed between iNPH and healthy controls were examined further, where iNPH was compared with AD, MCI/AD, MCI, and FTD. Linear regression was used to assess the association between protein level (NPX) and patient group, adjusting for the plate number (three different plates were used), age, and sex. The proteins were analyzed one at a time. Linear regression was assessed using analysis of variance (ANOVA) F-tests, and groups were compared in pairwise post hoc tests using t statistics. 95% confidence intervals for the log_2_ fold-changes are reported in the tables. Benjamini-Hochberg’s method for controlling the false discovery rate (FDR) was applied to adjust for the multiple tests performed. An FDR below 10% was considered significant. Correlations between protein measurements were assessed using Spearman’s correlation. A multivariate Partial Least-Squares Discriminant Analysis model (PLS-DA), based on proteins that differ between iNPH and healthy controls, was constructed to distinguish between iNPH and the other patient groups (AD, MCI/AD, MCI, FTD). The PLS-DA model’s performance was evaluated using 10 fivefold cross-validations.

## Results

### Study participants

The median age of all study participants was 74 (range 50–88), of whom 59% were men, and 41% were women. Patients with FTD and MCI/AD were slightly younger than the iNPH-patients (p < 0.01 and p < 0.05), while the controls were older (p < 0.001), see Table 1. There were no differences between the groups regarding sex distribution. The CSF biomarkers analyzed as part of the routine work-up are presented in Table 1. Levels of T-tau were lower in iNPH compared with all other groups, p < 0.001, while levels of amyloid beta 1–42 (Aß-42) were lower in iNPH than in controls, FTD, and MCI (p < 0.01, < 0.001 and < 0.001, respectively) but higher in iNPH than in patients with AD and MCI/AD (p < 0.001), see Table 1.

**Fig. 1:**
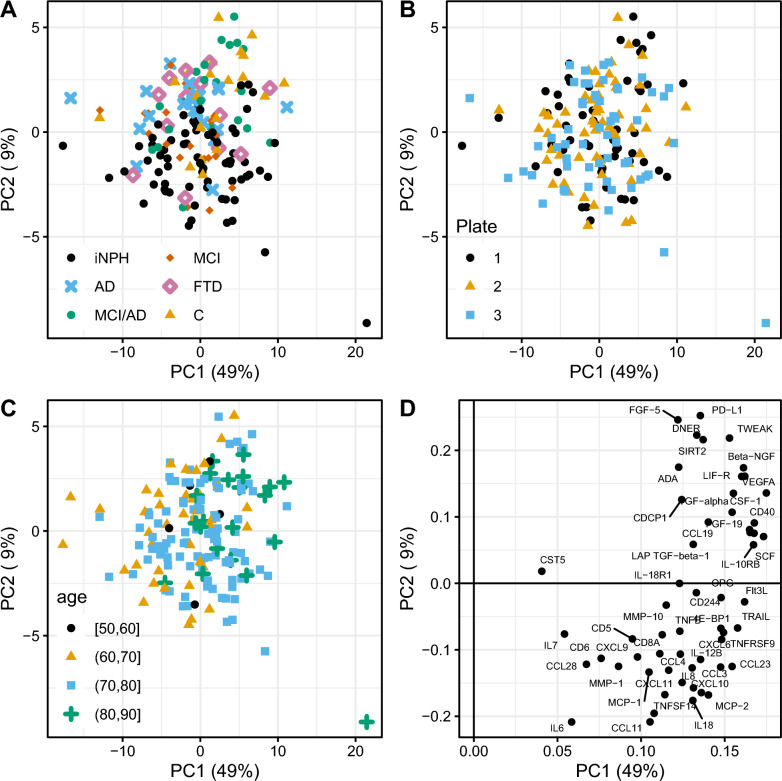
Principal component analysis (PCA) based on levels of 56 cerebrospinal fluid proteins. The score plot is colored according to (**A**) patient groups, (**B**) plate number (three plates were used) and (**C**) age. All included data is reduced into two dimensions. PC1 is the combination with the largest possible explained variation, PC2 is the second most important direction and orthogonal from PC1. Loading plot illustrated in (**D**) with proteins with higher levels in iNPH-patients in the bottom and lower levels in the top. *iNPH* idiopathic normal pressure hydrocephalus, *AD* Alzheimer’s disease, *MCI/AD* mild cognitive impairment due to Alzheimer’s disease, *MCI* mild cognitive impairment, *FTD* frontotemporal dementia and *C* controls

The PCA of all 56 analyzed proteins showed no apparent differences between analyzed plates or age groups (Fig. 1). There was a systematic trend regarding the difference in protein levels in iNPH-patients compared to other groups (Fig. 1A), as an example, illustrated by higher levels of CCL11 and lower levels of PD-L1 in iNPH (Fig. 1D).

### Difference between iNPH and healthy subjects.

**Fig. 2:**
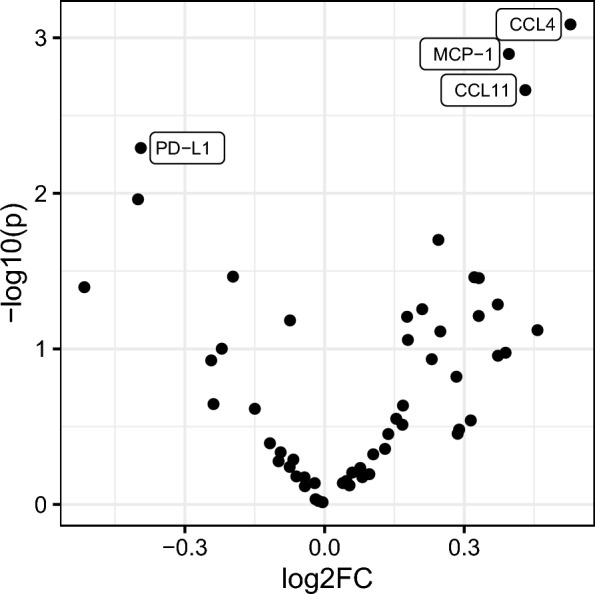
Volcano plot including the 56 cerebrospinal fluid proteins that had detectable values for at least 75% of the samples. The plot shows p-values (log10) and the magnitude of change (FC = fold change (log2)). The proteins that differed significantly (with false discovery rate 10%) between iNPH and controls are labeled. The analysis was adjusted for age, sex, and plate number

In a linear regression model adjusted for age, sex, and plate, the levels of four proteins differed significantly between patients with iNPH and healthy controls. Levels of chemokine ligand 4 (CCL4), monocyte chemoattractant protein 1 (MCP-1), and chemokine ligand 11 (CCL11) were higher in iNPH than in controls (p = 0.0008, p = 0.0013 and p = 0.0022, respectively). Programmed death ligand 1 (PD-L1) was lower in iNPH than in controls, p = 0.0051 (Table 2). Associations between protein level and diagnostic group, as well as age, sex, and plate for all analyzed proteins, are presented in Additional file 1: Table S1. To illustrate the difference between iNPH and controls for all proteins, the level of significance versus the fold change is visualized in a volcano plot (Fig. 2), and the difference is displayed in a box plot (Additional file 1: Figure S1).

**Table 2:** Associations between protein value (expressed in NPX) and diagnosis of iNPH when compared to healthy controls

*Porotein*	*Fold change (Log2)*	*p-value*	*q-value*
CCL4	0.528 (0.225, 0.831)	0.00082	0.036
MCP-1	0.396 (0.160, 0.632)	0.0013	0.036
CCL11	0.431 (0.160, 0.702)	0.0022	0.041
PD-L1	− 0.395 (− 0.668, − 0.122)	0.0051	0.072

The four analyzed proteins that differed significantly (10% FDR) between iNPH and healthy individuals, adjusted for age, sex and plate number. 95% confidence interval in the parenthesis. Q-values calculated using Benjamini Hochberg’s method for controlling the false discovery rate

### Differences between iNPH and neurodegenerative disorders

**Fig. 3:**
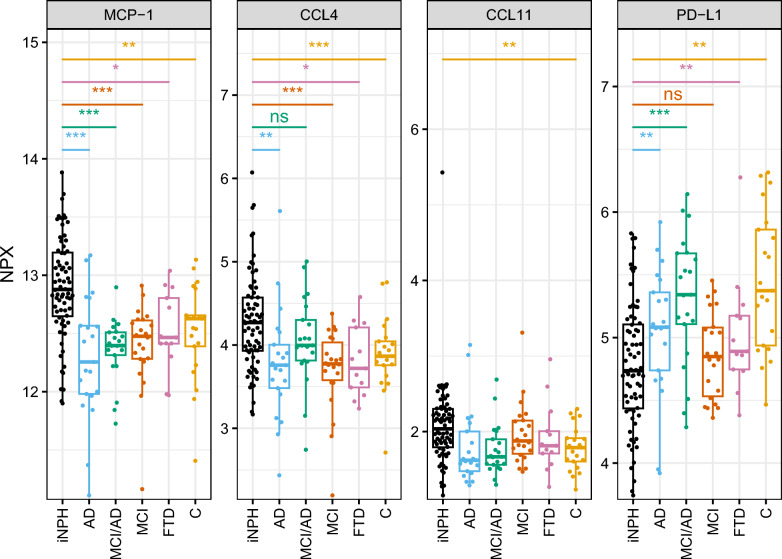
Box-plot including only the four proteins that differed between iNPH and controls in comparison to the other groups. P-values: *** < 0.001, ** < 0.01, * < 0.05, ns: non-significant. *iNPH* idiopathic normal pressure hydrocephalus, *AD* Alzheimer´s disease, *MCI/AD* mild cognitive impairment due to Alzheimer’s disease, *MCI* mild cognitive impairment, *FTD* frontotemporal dementia, *C* controls

Only the four proteins (MCP-1, CCL4, CCL11, and PDL-1) that differed between controls and iNPH were compared between iNPH and the other groups. MCP-1 was higher in iNPH than in all other patient groups. CCL4 was higher in iNPH than in all other groups except MCI/AD, while PD-L1 was lower in iNPH than all other groups, except MCI. There was no difference in levels of CCL11 between iNPH and other groups (Fig. 3). Absolute fold change and p-values for all comparisons between iNPH and the other groups are presented in Additional file 1: Table S2.

**Fig. 4:**
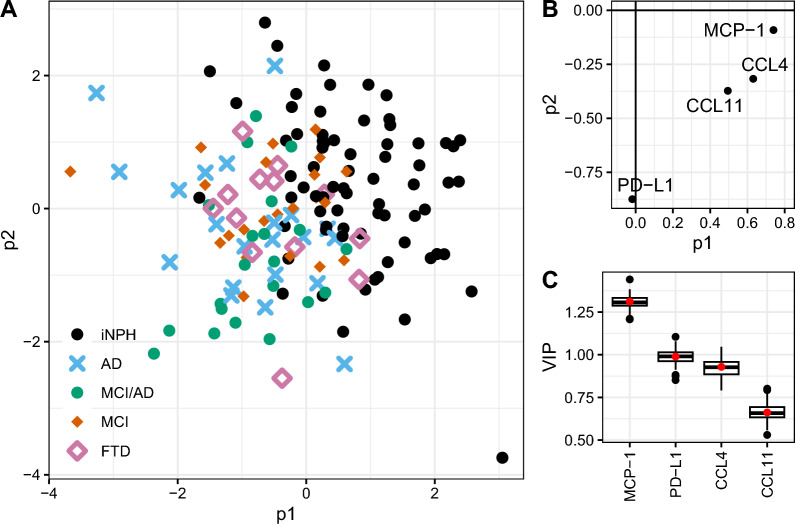
To investigate if a model of the four proteins MCP-1, CCL4, PD-L1 and CCL11 could predict diagnosis of iNPH vs AD, MCI/AD, MCI or FTD, a PLS-DA multivariate model was built. **A**. PLS-DA score plot. **B**. Loading plot with the four proteins. **C**. The VIP (variable of importance) values illustrates the predictive importance of a variable in the model. The red dots represent the VIP for the full model (based on all data). The boxplots represent the VIP for the cross validated models

A PLS-DA model was created to investigate whether a combination of the four proteins (MCP-1, CCL4, PD-L1, and CCL11) could be used to identify iNPH versus the other patient groups. The mean area under the ROC curve of the model to discriminate between iNPH and the other groups was 0.91, with a mean error rate of 0.18 (mean computed over the 50 test sets). A high variable importance (VIP) value for a protein indicates that the protein contributes greatly to the divergence between the diagnostic groups. The VIP of MCP-1 was the highest among the analyzed proteins (> 1.25), see Fig. 4.

### Correlation between proteins and age

CCL4 and CCL11 showed a moderate correlation to MCP-1 and a weak correlation to PD-L1. PD-L1 was consistently but weakly correlated with the other three proteins (Additional file 1: Figure S2). Of the 56 analyzed proteins, there were associations between age and protein levels in 36 proteins, Additional file 1: Table S1. Of the four significant proteins (MCP-1, CCL4, PD-L1 and CCL11), there were associations between age and protein levels for all but MCP-1.

## Discussion

The study analyzed 56 proteins in CSF related to inflammation, by proximity extension assay in patients with iNPH, healthy controls, and groups with relevant differential diagnoses. Four proteins differed between iNPH and healthy controls, and out of these, MCP-1, CCL4, and PD-L1 also differed between iNPH and the differential diagnoses. A predictive model based on all four proteins with the aim to discriminate between iNPH and the groups with differential diagnoses had a high mean AUC of 0.91.

Markers of neuroinflammation have been reported in neurodegenerative diseases and secondary hydrocephalus; such as posthemorrhagic hydrocephalus, where upregulation of CCL proteins and interleukins have been reported [14]. Previous studies of neuroinflammation in iNPH are limited, but in a recent study using the same PEA assay as the one in this study, the levels of 12 cytokines differed between iNPH and healthy controls [15]. None of those cytokines were MCP-1, CCL4, CCL11, or PD-L1. However, they did not control for multiple analyses and age, and in our study, the levels of 64% of proteins were age-dependent. Another study using proteomic analysis of CSF biomarkers has shown correlation to clinical improvement in shunt response [16].

### MCP-1 involved in activation of many cell types in choroid plexus

MCP-1 is involved in inflammation by activation of monocytes/macrophages and recruitment of monocytes, microglia, and T helper cells. This cytokine is produced by many cell types, such as fibroblasts, and endothelial, epithelial, smooth muscle, mesangial, astrocytic, monocytic, and microglial cells [17–20]. It is associated with oxidative stress, and could therefore indirectly affect progression in several diseases such as atherosclerosis, diabetes, and AD [21–23]. In the brain, MCP-1 is responsible for recruitment and accumulation of leucocytes in the choroid plexus, [24] a structure responsible for CSF secretion through membrane transport mechanisms [25]. In line with our results, Jeppsson et al., using a different method, reported elevated MCP-1 in the CSF of iNPH patients compared with different groups of neurodegenerative diseases [26]. Levels of MCP-1 are also increased in human traumatic brain injury, [27] and are also reported to increase in plasma in early stages of AD but then decrease in later stages [28]. The increased MCP-1 levels in the prodromal phase of AD have also been shown in a review study and correlated to a faster cognitive decline [29]. Whether levels of MCP-1 also change during different stages of iNPH remains to be investigated.

### CCL4 involved in dysfunction of the blood brain barrier (BBB)

CCL4 is upregulated in several neurological disorders such as multiple sclerosis, Parkinson’s disease, and AD [30–32]. In the present study, CCL4 was higher in iNPH than in all other investigated groups, except MCI/AD. This indicates that it could be an unspecific marker that is upregulated in response to CNS injury. It is mainly secreted by macrophages and is an essential chemoattractant for inflammatory cells [33, 34]. It is reported to have an effect on brain endothelial cells and therefore contributes to the dysfunction of BBB [35, 36]. Levels of CCL4 were increased in post-hemorrhagic hydrocephalus in a recent study, [14] but to our knowledge it has not previously been reported in iNPH.

### CCL11 in traumatic brain injury and iNPH

CCL11 is known to play a role in eosinophilic and basophilic activities, often linked to inflammatory allergic reactions such as asthma, atopic dermatitis, and inflammatory bowel disease [37, 38]. Our study shows an elevation of CCL11 levels in iNPH when compared to the control group, yet no significant differences were observed in comparison with neurodegenerative diagnoses. There is a lack of previous reports linking CCL11 to the context of iNPH, and no documented associations between CCL11 levels and reduced walking ability or cognitive dysfunction. However, a recent cohort study involving brain tissue analysis via ELISA demonstrated elevated CCL11 levels in the CSF of football players with traumatic encephalopathy. Remarkably, these elevated levels were also found to be correlated with years of exposure to American football [39]. This raises the possibility of potential shared mechanisms between traumatic brain injury and iNPH, possibly involving neuroinflammatory processes.

### PD-L1 involved in antitumor activity

PD-L1 binds to programmed cell death 1 receptor (PD-1), attached to T and B cells, macrophages, and dendritic cells. It is primarily expressed in parenchymal organs such as the heart, placenta, skeletal muscle, and lungs, and is upregulated in blood and tumors in various cancers [40, 41]. It mediates inhibitory signals to T-cell activation and suppresses cell inflammation, and through this action escapes the antitumor response [42, 43]. To our knowledge, there are no previous reports of divergent levels of PD-L1 in the CSF of patients with iNPH, but in a recent study elevated levels of PD-L1 were seen in MCI/AD compared to the MCI group, with decreased levels in FTD compared to controls [13]. This is interesting, because in the present study PD-L1 was lower in iNPH than in all other groups but MCI. Note that data from the neurodegenerative groups [13] was compared with the iNPH patients in the present study.

### Strengths

The strengths of this study are that all CSF samples were collected at a single center, which strengthens credibility of the analysis. Plate number and age were also adjusted for in the statistical analyses, which is important, since a majority of the analyzed proteins are age dependent. Furthermore, all CSF samples were analyzed at the same time, which reduces error sources. PEA and a similar method—proximity ligation assays—have reported higher sensitivity and specificity compared to previous ELISA methods [44–46]. CSF biomarkers in iNPH are usually somewhat lower compared to controls and neurodegenerative conditions [26]. Therefore, the higher levels of some of these analyzed proteins in iNPH in this study indicate a true difference.

## Limitations

Limitations include the diagnostic uncertainty in groups with neurodegenerative disorders where follow-up time confirms the diagnoses. Comorbidity with AD is common in iNPH, [2, 47] and we could not completely exclude whether the iNPH patients had ongoing AD development or would develop it over the long term. While disease duration could potentially influence the levels of the measured proteins, it would have been advantageous to incorporate this variable into our statistical analyses. Nonetheless, we opted not to include disease duration due to the considerable variation in how individuals perceive the onset of symptoms, rendering it a very unreliable factor.

Another limitation is the small sample sizes in some of the neurodegenerative groups that may have influenced the accuracy in the results. Furthermore, the large number of analyses in relation to the low amount of patient material could produce false positive results, therefore a validation with another cohort analysis would be of value. However, corrections for multiple comparisons were performed. In our study, we specifically focused on patients with FTD or at different stages of AD. However, it is important to note that conditions like vascular dementia and neurodegenerative disorders such as Lewy body dementia, progressive supranuclear palsy, and multiple-system atrophy can also exhibit symptoms resembling those of iNPH [48]. Regrettably, these diagnostic categories were not included in our study, which limits the study's ability to definitively draw conclusions regarding the diagnostic potential of the biomarkers. Nonetheless, Jeppson et al. did incorporate these diagnoses in their study and found outcomes concerning MCP-1 consistent with our findings [26].

## Conclusion

Using proximity extension assay, two inflammatory cytokines (MCP-1 and CCL4) were increased, and one (PD-L1) was reduced in patients with iNPH compared with Alzheimer’s disease, mild cognitive impairment, and frontotemporal dementia. Neuroinflammation could play a role in the mechanisms of iNPH, and cytokines such as MCP-1, CCL4, and PD-L1 may possibly have diagnostic potential in the work-up of patients with iNPH.

### Supplementary Information

**Additional file 1: Table S1**. Association between all analyzed proteins (expressed in NPX) and groups (iNPH versus C). All 56 analyzed proteins are compared with iNPH and healthy individuals, adjusted for age, sex, and plate. **Figure S1**. The four proteins that differ significantly between iNPH and controls. **Table S2**. Association between the selected proteins and neurodegenerative groups. **Figure S2**. Spearman correlations between the four selected proteins. **Figure S3.** Spearman correlations between the four selected proteins.

## Data Availability

The datasets analyzed during the current study are available from the corresponding author on reasonable request.
