# Supplementary material for: Levels of inflammatory cytokines MCP-1, CCL4, and PD-L1 in CSF differentiate idiopathic normal pressure hydrocephalus from neurodegenerative diseases
Source: Fluids Barriers CNS. 2023 Oct 13;20:72. doi: 10.1186/s12987-023-00472-x (PMC10571396; doi:10.1186/s12987-023-00472-x)
Supplement: Supplementary file 1 — Additional file 1: Table S1. Association between all analyzed proteins (expressed in NPX) and groups (iNPH versus C). All 56 analyzed proteins are compared with iNPH and healthy individuals, adjusted for age, sex, and plate. Figure S1. The four proteins that differ significantly between iNPH and controls. Table S2. Association between the selected proteins and neurodegenerative groups. Figure S2. Spearman correlations between the four selected proteins. Figure S3. Spearman correlations between the four selected proteins. [file 12987_2023_472_MOESM1_ESM.docx]

**Additional file**

**Additional file 1: Table S1.** Association between all analyzed proteins (expressed in NPX) and groups (iNPH versus C). All 56 analyzed proteins are compared with iNPH and healthy individuals, adjusted for age, sex, and plate.

| **Protein** | **log2FC NPH-C** | **p-value NPH-C** | | **q-value NPH-C** | **p-value Age** | **p-value Male** | **p-value Plate** |
| --- | --- | --- | --- | --- | --- | --- | --- |
| CCL4 | 0.528 (0.225, 0.831) | | 0.000822 | 0.0356 | 0.0327 | 0.231 | 0.0477 |
| MCP-1 | 0.396 (0.160, 0.632) | | 0.00127 | 0.0356 | 0.4 | 0.00231 | 0.205 |
| CCL11 | 0.431 (0.160, 0.702) | | 0.00217 | 0.0405 | 0.0162 | 0.0152 | 0.268 |
| PD-L1 | –0.395 (–0.668, –0.122) | | 0.00512 | 0.0716 | 0.000202 | 0.886 | 0.0928 |
| FGF-5 | -0.401 (-0.707, -0.094) | | 0.0109 | 0.122 | 0.000217 | 0.328 | 0.275 |
| TNFSF14 | 0.244 (0.040, 0.449) | | 0.0199 | 0.186 | 0.306 | 0.626 | 0.404 |
| Beta-NGF | –0.197 (–0.378, –0.015) | | 0.0343 | 0.218 | 0.000327 | 0.224 | 0.629 |
| IL18 | 0.322 (0.024, 0.619) | | 0.0347 | 0.218 | 0.149 | 0.0378 | 0.791 |
| CCL3 | 0.331 (0.024, 0.639) | | 0.0351 | 0.218 | 0.0789 | 0.00431 | 0.0985 |
| CCL19 | –0.516 (–1.008, –0.024) | | 0.0401 | 0.225 | 0.0408 | 0.00091 | 0.49 |
| MCP-2 | 0.372 (–0.003, 0.747) | | 0.0518 | 0.245 | 0.147 | 0.0111 | 0.713 |
| Flt3L | 0.210 (–0.005, 0.425) | | 0.0556 | 0.245 | 0.00401 | 0.031 | 0.666 |
| CCL23 | 0.331 (–0.016, 0.678) | | 0.0615 | 0.245 | 0.00022 | 0.00589 | 0.387 |
| IL7 | 0.177 (–0.009, 0.364) | | 0.0622 | 0.245 | 0.00527 | 0.617 | 0.399 |
| DNER | –0.074 (–0.153, 0.005) | | 0.0656 | 0.245 | 0.00458 | 0.0641 | 0.329 |
| MCP-4 | 0.457 (–0.049, 0.963) | | 0.0758 | 0.255 | 0.00932 | 0.0316 | 0.427 |
| IL8 | 0.249 (–0.028, 0.525) | | 0.0773 | 0.255 | 0.173 | 0.122 | 0.803 |
| CCL28 | 0.179 (–0.027, 0.385) | | 0.0876 | 0.272 | 0.0942 | 0.655 | 0.102 |
| TWEAK | –0.221 (–0.484, 0.043) | | 0.0997 | 0.289 | 1.83e–05 | 0.788 | 0.318 |
| CXCL11 | 0.389 (–0.084, 0.862) | | 0.106 | 0.289 | 0.217 | 0.841 | 0.609 |
| CXCL5 | 0.372 (–0.087, 0.832) | | 0.111 | 0.289 | 0.00823 | 0.179 | 0.526 |
| IL6 | 0.230 (–0.058, 0.519) | | 0.117 | 0.289 | 0.159 | 0.0092 | 0.36 |
| ADA | –0.243 (–0.550, 0.064) | | 0.119 | 0.289 | 0.0597 | 0.35 | 0.186 |
| 4E-BP1 | 0.283 (–0.105, 0.672) | | 0.151 | 0.352 | 0.00621 | 0.207 | 0.969 |
| FGF-19 | –0.239 (–0.628, 0.151) | | 0.226 | 0.499 | 0.00356 | 0.258 | 0.272 |
| TNFB | 0.168 (–0.109, 0.446) | | 0.232 | 0.499 | 0.0719 | 0.6 | 0.648 |
| CDCP1 | –0.150 (–0.403, 0.103) | | 0.243 | 0.504 | 0.00409 | 0.894 | 0.497 |
| CD5 | 0.154 (–0.128, 0.436) | | 0.281 | 0.557 | 0.149 | 0.39 | 0.652 |
| CXCL10 | 0.314 (–0.270, 0.898) | | 0.288 | 0.557 | 0.277 | 0.318 | 0.817 |
| IL-12B | 0.167 (–0.156, 0.491) | | 0.307 | 0.574 | 0.122 | 0.514 | 0.983 |
| MMP-1 | 0.289 (–0.298, 0.876) | | 0.33 | 0.597 | 0.0524 | 0.187 | 0.45 |
| CXCL9 | 0.286 (–0.320, 0.892) | | 0.351 | 0.599 | 6.94e–05 | 0.306 | 0.636 |
| OPG | 0.137 (–0.154, 0.428) | | 0.353 | 0.599 | 5.06e–05 | 0.000338 | 0.522 |
| VEGFA | –0.117 (–0.396, 0.161) | | 0.404 | 0.666 | 0.000585 | 0.341 | 0.22 |
| CXCL1 | 0.130 (–0.202, 0.462) | | 0.439 | 0.703 | 0.0494 | 0.171 | 0.801 |
| SIRT2 | –0.094 (–0.348, 0.160) | | 0.462 | 0.719 | 0.00132 | 0.156 | 0.318 |
| HGF | 0.104 (–0.186, 0.395) | | 0.476 | 0.721 | 0.000143 | 0.487 | 0.849 |
| CSF-1 | –0.067 (–0.271, 0.137) | | 0.515 | 0.758 | 0.000267 | 0.275 | 0.771 |
| CX3CL1 | –0.099 (–0.408, 0.211) | | 0.528 | 0.758 | 0.00112 | 0.197 | 0.882 |
| CD40 | –0.075 (–0.338, 0.189) | | 0.574 | 0.796 | 0.000899 | 0.17 | 0.542 |
| TRAIL | 0.077 (–0.200, 0.353) | | 0.583 | 0.796 | 0.0324 | 0.323 | 0.69 |
| CD6 | 0.059 (–0.180, 0.299) | | 0.624 | 0.802 | 0.709 | 0.451 | 0.442 |
| CD8A | 0.096 (–0.310, 0.502) | | 0.639 | 0.802 | 0.658 | 0.03 | 0.808 |
| TNFRSF9 | –0.061 (–0.334, 0.213) | | 0.66 | 0.802 | 0.196 | 0.0034 | 0.535 |
| MMP-10 | 0.081 (–0.293, 0.455) | | 0.668 | 0.802 | 0.0592 | 0.0583 | 0.496 |
| LIF-R | –0.043 (–0.243, 0.157) | | 0.67 | 0.802 | 0.000574 | 0.12 | 0.433 |
| CD244 | 0.047 (–0.200, 0.294) | | 0.707 | 0.802 | 0.000123 | 0.00157 | 0.849 |
| CCL25 | 0.045 (–0.203, 0.294) | | 0.718 | 0.802 | 0.000732 | 5.17e–05 | 0.509 |
| IL-10RB | 0.039 (–0.185, 0.264) | | 0.729 | 0.802 | 6.78e–05 | 0.0403 | 0.385 |
| CST5 | –0.021 (–0.142, 0.100) | | 0.73 | 0.802 | 0.289 | 0.0608 | 0.251 |
| uPA | 0.046 (–0.217, 0.309) | | 0.73 | 0.802 | 0.000391 | 0.347 | 0.699 |
| IL-18R1 | 0.054 (–0.285, 0.392) | | 0.754 | 0.807 | 0.0345 | 0.684 | 0.964 |
| SCF | –0.042 (–0.321, 0.237) | | 0.764 | 0.807 | 0.000605 | 0.00804 | 0.961 |
| LAP TGF-beta-1 | –0.019 (–0.433, 0.394) | | 0.926 | 0.96 | 0.0063 | 0.815 | 0.858 |
| CXCL6 | –0.013 (–0.417, 0.391) | | 0.95 | 0.967 | 0.0341 | 0.00789 | 0.406 |
| TGF-alpha | –0.004 (–0.214, 0.205) | | 0.968 | 0.968 | 0.0098 | 0.16 | 0.771 |

**Additional file 1: Figure S1.** The four proteins that differ significantly between iNPH and controls.

| **Protein** | **p.ANOVA** | **Patient groups** | **Log_2_FC** | **p-value** |
| --- | --- | --- | --- | --- |
| CCL11 | 0.181 | NPH-FTD |  |  |
| CCL11 | 0.181 | NPH-AD |  |  |
| CCL11 | 0.181 | NPH-MCI/AD |  |  |
| CCL11 | 0.181 | NPH-MCI |  |  |
| CCL4 | 0.000478 | NPH-FTD | 0.360 (0.701, 0.019) | 0.0389 |
| CCL4 | 0.000478 | NPH-AD | 0.446 (0.722, 0.171) | 0.00169 |
| CCL4 | 0.000478 | NPH-MCI/AD | 0.162 (0.440, –0.116) | 0.252 |
| CCL4 | 0.000478 | NPH-MCI | 0.530 (0.801, 0.259) | 0.000165 |
| MCP-1 | 1.04e–08 | NPH-FTD | 0.310 (0.560, 0.060) | 0.0155 |
| MCP-1 | 1.04e–08 | NPH-AD | 0.563 (0.765, 0.361) | 1.66e-07 |
| MCP-1 | 1.04e–08 | NPH-MCI/AD | 0.487 (0.691, 0.283) | 5.54e–06 |
| MCP-1 | 1.04e–08 | NPH-MCI | 0.467 (0.665, 0.268) | 7.59e–06 |
| PD-L1 | 8.36e–06 | NPH-FTD | –0.388 (–0.106, –0.670) | 0.00743 |
| PD-L1 | 8.36e–06 | NPH-AD | –0.329(–0.101, –0.557) | 0.00494 |
| PD-L1 | 8.36e-06 | NPH-MCI/AD | –0.601 (–0.371, –0.831) | 7.88e–07 |
| PD-L1 | 8.36e–06 | NPH-MCI | –0.129 (0.094, –0.353) | 0.255 |

**Additional file 1: Table S2**. Association between the selected proteins and neurodegenerative groups.

**Additional file 1: Figure S2.** Spearman correlations between the four selected proteins.

**Additional file 1: Figure S3.** Spearman correlations between the four selected proteins.
